# Constitutive overexpression of the pollen specific gene *SKS13* in leaves reduces aphid performance on *Arabidopsis thaliana*

**DOI:** 10.1186/s12870-014-0217-3

**Published:** 2014-08-14

**Authors:** Xi Chen, Zhao Zhang, Richard G F Visser, Ben Vosman, Colette Broekgaarden

**Affiliations:** Wageningen UR, Plant Breeding, PO. Box 386, Wageningen, 6700 AJ the Netherlands; Laboratory of Phytopathology, Wageningen University, Droevendaalsesteeg 1, 6708PB, Wageningen, the Netherlands

**Keywords:** Activation tag mutant, *Brevicoryne brassicae*, Electrical penetration graph, Jasmonic acid, *Myzus persicae*, Phloem-feeding insect, Reactive oxygen species

## Abstract

**Background:**

Plants have developed a variety of mechanisms to counteract aphid attacks. They activate their defences by changing the expression of specific genes. Previously we identified an activation tag mutant of *Arabidopsis thaliana* on which *Myzus persicae* population development was reduced. Activation tag mutants are gain-of-function in which the expression of a gene is increased by the insertion of the *Cauliflower mosaic virus* 35S enhancer that acts on the natural promoter. By further characterizing this previously identified mutant we identified a gene that reduces performance of *M. persicae* and also provided clues about the mechanism involved.

**Results:**

We show that *SKU5 SIMILAR 13* (*SKS13*), a gene whose expression in wild type plants is restricted to pollen and non-responsive to *M. persicae* attack, is overexpressed in the *A. thaliana* mutant showing reduced performance of *M. persicae*. Monitoring *M. persicae* feeding behaviour on *SKS13* overexpressing plants indicated that *M. persicae* have difficulties feeding from the phloem. The constitutive expression of *SKS13* results in accumulation of reactive oxygen species, which is possibly regulated through the jasmonic acid pathway. The enhanced resistance is not aphid species specific as also the population development of *Brevicoryne brassicae* was affected*.*

**Conclusions:**

We demonstrate that constitutive expression in leaves of the pollen-specific gene *SKS13* can enhance plant defence, resulting in a reduction of *M. persicae* population development and also decreases the transmission of persistent viruses. Overexpression of *SKS13* in *A. thaliana* also affects *B. brassicae* and possibly other phloem feeding insects as well. Identifying genes that can enhance plant defence against insects will be important to open up new avenues for the development of insect resistant crop plants.

## Background

Aphids have a sophisticated feeding strategy in which they use their stylets to penetrate plant tissue and puncture cells along the intercellular pathway towards the phloem [1]. To facilitate the probing and feeding processes, aphids secrete saliva into the plant tissue to degrade cell walls and to overcome occlusion of the feeding site [2,3]. Once an aphid establishes a feeding site it can feed from the phloem of a susceptible plant for hours or even days [1]. Aphid infestation limits plant productivity due to the depletion of photo-assimilates and the deposition of excess sugars as honeydew that encourages growth of mold. In addition, aphids are important vectors of numerous plant viruses that can be transmitted during probing and feeding, resulting in additional damage to plants [4].

Plants have evolved a series of defense traits to directly affect the aphid’s feeding behavior. These defenses include physical and chemical traits that can be constitutively present or induced upon aphid attack [5]. Physical traits, such as hairs and glandular trichomes, hinder aphid settling on a plant [6]. Chemical traits include the production of secondary metabolites and proteins that are repellent or toxic to aphids thereby affecting their performance [7]. For example, the brassicaceous-specific secondary metabolites glucosinolates have been shown to negatively affect the performance of the generalist aphid *Myzus persicae* [8]. Contrary to constitutive traits, inducible defenses require recognition of the attacking aphid and subsequent transcriptional reprogramming. This also includes the activation of general wound responses. An increasing body of evidence suggests that reactive oxygen species (ROS), which were always thought to be induced as a general wound response, can play a role in plant defense towards aphids as well [9,10]. For example, an early accumulation of ROS upon Russian wheat aphid infestation was suggested to be a defense response in aphid resistant wheat [11]. In contrast, an increasing concentration of ascorbic acid, a compound that is capable of reducing ROS, leads to an enhanced aphid fecundity [10], further underpinning the role of ROS in plant defense towards aphids. Moreover, ROS can act as signaling molecules, along with JA, to confer aphid resistance [12]. The activation of plant hormone pathways, especially jasmonic acid (JA), salicylic acid (SA) and ethylene (ET), plays an important role in plant defense against aphids [13,14]. These pathways interact in a network, regulating the expression of specific groups of defense-related genes [15]. Although all pathways can be involved in defense, the JA pathway is thought to be the most effective against aphids [16,17]. Constitutive activation of the JA pathway in an *Arabidopsis thaliana* mutant leads to enhanced aphid resistance, whereas blocking the JA pathway results in aphid susceptibility [14].

It has been shown that certain genes, for instance *IQD1* (*IQ-Domain1*) and *MPL1* (*Myzus persicae –induced lipase 1*) can confer plant resistance to insects when their level of expression is increased or the location of expression is changed [18–20]. Such genes may be identified by screening activation tag mutant collections for insect resistance [18,21]. In these mutants, tagged genes are overexpressed by a tetramer *Cauliflower mosaic virus* (CaMV) 35S enhancer adjacent to the natural promoter, resulting in a dominant gain-of-function phenotype [22]. By screening such a mutant collection of *A. thaliana*, we have identified several mutants with enhanced resistance against *M. persicae* [23]. In the present paper we characterize one of these mutants, leading to the identification of *SKU5 SIMILAR 13* (*SKS13*) as a gene responsible for enhanced resistance to *M. persicae*. We analyzed the feeding behavior of *M. persicae* on the mutant using the electrical penetration graph (EPG) technique [24] to get information about the location of resistance factors. Based on the putative involvement of *SKS13* in oxidation/reduction reactions we visualized the accumulation of ROS in leaves. Finally, we monitored the expression of several JA-, SA, and ethylene-pathway marker genes to study the possible interaction of *SKS13* with these hormone pathways that may explain the aphid resistance conferred by *SKS13* overexpression.

## Results

### Phenotypic characterization of mutant 3790

**Figure 1:**
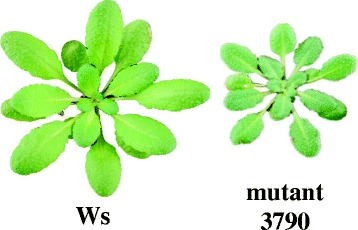
**Phenotype of** ***A. thaliana*** **mutant 3790.** Rosette leaf phenotype of six-week old Wassilewskija (Ws) and mutant 3790.

Mutant 3790 was previously identified as an *Arabidopsis thaliana* activation tag mutant on which *Myzus persicae* shows a longer pre-reproductive period and produces smaller numbers of offspring than on its corresponding wild type Wassilewskija (Ws) [23]. Compared to Ws, mutant 3790 has smaller and darker green colored leaves (Figure 1), shows a delayed flowering, a reduced height of the main stem and an increased number of lateral branches.

### Identification of *SKS13* as a gene conferring enhanced resistance to *M. persicae*

**Figure 2:**
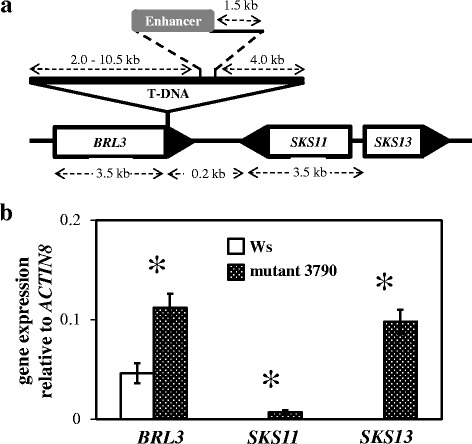
**Location of the activation tag and expression analysis. (a)** Genomic region of mutant 3790 showing the T-DNA insert containing the CaMV35S enhancer. The T-DNA is located in the 3’-UTR (black triangle) of *BRL3*. The exact distance between the adjacent genes *SKS11* and *SKS13* with their promoters and the enhancer is unknown. Diagram is not drawn to scale. **(b)** Quantitative RT-PCR expression analysis of *BRL3*, *SKS11* and *SKS13* in rosette leaves of Ws and mutant 3790. Values are the means ± SD (n = 3). The star indicates a significant difference between bars within a pair (Independent-samples *t*-test, *P* < 0.05).

Using inverse PCR we could determine that mutant 3790 contains a T-DNA including a 35S enhancer that is located on chromosome 3 at position 4,350,852 (according to the TAIR website; http://www.arabidopsis.org) in the 3’-UTR region of the *Brassinosteroid Receptor Like* gene (*BRL3*, At3g13380; Figure 2a). Additionally, two other genes, *SKU5 Similar 11* (*SKS11*, At3g13390) and *SKU5 Similar 13* (*SKS13*, At3g13400) are located within a distance of approximately 8 kb of the enhancer (Figure 2a), a distance over which the enhancer can effectively activate the expression of genes [25]. To determine whether the transcript levels of these three genes were affected by the enhancer, we first performed quantitative RT-PCR (qPCR). The transcript level of *BRL3* was two-fold higher in mutant 3790 than in Ws (Figure 2b). No transcripts of *SKS11* and *SKS13* were detectable in Ws but they could clearly be detected in mutant 3790 (Figure 2b).

As *A. thaliana* knockout mutants for many genes are publically available, we determined whether impaired expression of *BRL3* affects the performance of *M. persicae*. To this purpose, we performed no-choice aphid assays and compared *M. persicae* population development on *BRL3* knockout mutants *brl3-2* and *brl3-3* [26] with that on wild type Columbia-0 (Col-0). The numbers of *M. persicae* on these mutants (18.5 ± 5.6 on *brl3-2* and 16.2 ± 4.1 on *brl3-*3) did not differ from that on Col-0 (19.5 ± 7.0; Kruskal–Wallis followed by Mann–Whitney *U* test, *P* > 0.05, n = 15). Because *SKS11* and *SKS13* are not expressed in control leaves of Ws plants (Figure 2b), we performed a qPCR experiment to reveal whether these genes are induced upon infestation by *M. persicae*. Induced expression of *Lipoxygenase 2* (*LOX2*; data not shown) indicated an efficient infestation of *M. persicae* [27], but the expression of *SKS11* and *SKS13* remained undetectable in Ws leaves six and 24 hours after infestation of *M. persicae*. Therefore, we did not evaluate *M. persicae* performance on *SKS11* or *SKS13* knockout mutants.

**Figure 3:**
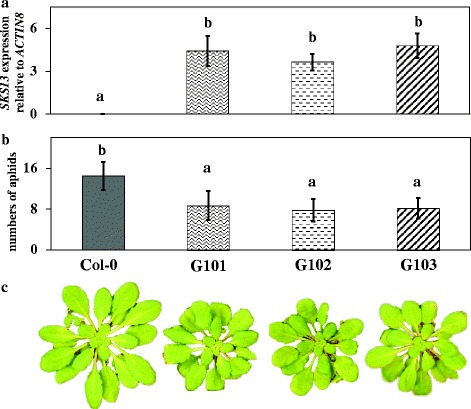
**Gene expression analysis,** ***Myzus persicae*** **aphid performance and phenotype of three independent** ***SKS13*** **overexpressing transgenic lines. (a)** Quantitative RT-PCR expression analysis of *SKS13* in rosette leaves of Columbia-0 (Col-0) and the three transgenic lines G101, G102 and G103. Values are the means ± SD (n = 3). **(b)** Performance of *M. persicae* on plants of Col-0 and the three transgenic lines G101, G102 and G103. Values are the means ± SD (n = 15). Bars marked with different letters are significantly different from each other (Kruskal–Wallis followed by Mann–Whitney *U* tests, *P* < 0.05). **(c)** Rosette leaf phenotype of six-week-old Col-0 and transgenic lines G101, G102 and G103.

Due to the orientation regarding the position of the transposon (Figure 2a) and strongest overexpression impact (Figure 2b), we decided to focus on *SKS13* for the continuation of this study. To confirm that overexpression of *SKS13* enhances resistance to *M. persicae*, we generated transgenic Col-0 lines (G101, G102 and G103) in which *SKS13* is overexpressed by the CaMV 35S promoter. Compared to Col-0, these lines showed significantly higher expression levels of *SKS13* (Figure 3a) and lower numbers of *M. persicae* (Figure 3b). Similar to mutant 3790, plants of these transgenic lines had smaller, rounder rosette leaves than their corresponding wild type (Figure 3c), and delayed flowering. The height of the main stem and the numbers of lateral branches of plants from these transgenic lines did not differ from Col-0.

### Feeding behavior of *M. persicae* on mutant 3790

To reveal whether aphid feeding behavior was affected by overexpression of *SKS13* we compared electrical penetration graph (EPG) [1] recordings of *M. persicae* on mutant 3790 and Ws plants. The EPG parameters relevant for our study are summarized in Table 1. No differences were observed for EPG parameters related to epidermal or xylem tissue. Also the total time of the pathway phase was similar on Ws and mutant 3790 (Table 1). *Myzus persicae* showed a significantly longer duration of the non-probing phase on mutant 3790 than on Ws (Table 1). Significant differences were also observed for pre-phloem and phloem phase-related activities. Compared to Ws, *M. persicae* on mutant 3790 needed double the amount of time to the first phloem phase, but spend only about one third of the total time in this phase (Table 1). Additionally, fewer *M. persicae* showed sustained phloem sap ingestion on mutant 3790 than on WS and the ones that did show this activity on mutant 3790 did this a smaller number of times (Table 1). Furthermore, aphids on Ws spent significantly more time salivating into the phloem and ingesting phloem sap than aphids on mutant 3790 (Table 1).

**Table 1:** **Electrical Penetration Graph (EPG) parameters considered and their relation to** ***Myzus persicae*** **feeding activity on** ***Arabidopsis thaliana*** **Ws and mutant 3790**

**Related tissue**	**EPG parameter**	**Wild type (Ws) n** ^**1**^ **= 18**	**Mutant 3790 n = 15**	***P*** **value** ^**2**^
Epidermal	Time to first probe (min)	2.5 ± 0.7	2 ± 0.4	0.870
Prephloem	Number of probes before first phloem contact	10.5 ± 2.6	13.2 ± 4.6	0.969
	Time from first probe to first phloem contact (min)	61.6 ± 12.4	113.6 ± 15.5	0.024
Phloem	Total time of phloem salivation (min)	10.8 ± 1.5	8.1 ± 1.6	0.025
	Number of phloem salivation events	14.3 ± 1.8	10.1 ± 1.8	0.240
	Average duration of phloem salivation (min)	0.8 ± 0.1	0.7 ± 0.1	0.462
	Total time of phloem ingestion (min)	97.5 ± 10.4	33.3 ± 8.2	0.001
	Number of phloem ingestion events	13.3 ± 1.6	8.7 ± 1.7	0.110
	Average duration of phloem ingestion (min)	7.8 ± 0.8	4.3 ± 1.0	0.003
	Total time of sustained (>10 min) phloem ingestion	64.2 ± 9.4	20.4 ± 2.5	0.002
	Number of sustained (>10 min) phloem ingestion	3.9 ± 0.6	1.2 ± 0.3	0.001
	Average duration of sustained (>10 min) phloem ingestion	17.4 ± 2.5	12.7 ± 1.7	0.032
All tissues	Total time of non-probing (min)	106.4 ± 16.9	148.3 ± 11.3	0.023
	Total time of pathway phase (min)	247.5 ± 11.5	261.4 ± 13.3	0.278
	Number of aphids with sustained (>10 min) phloem ingestion	18.0 (100%)	11.0 (73%)	0.030
Xylem	Total time of G	15.2 ± 5.8	16.2 ± 4.7	0.912
	Number of G	0.7 ± 0.3	0.3 ± 0.2	0.195

Values are means ± SE of EPG parameters during 8 h monitoring. ^1^EPG replicates; ^2^Mann Whitney U (duration) or Fisher exact (number) test *P* values.

### Accumulation of reactive oxygen species in mutant line 3790

**Figure 4:**
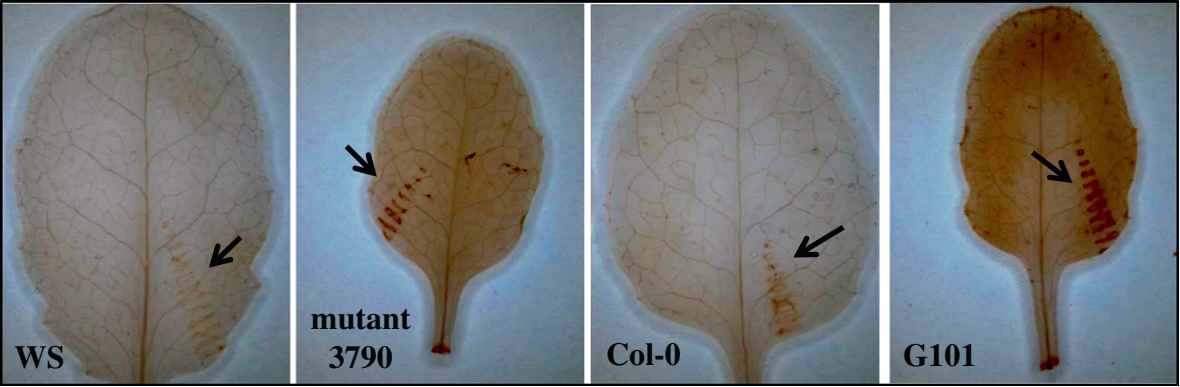
**Accumulation of reactive oxygen species (ROS) in** ***SKS13*** **overexpressing plants.** 3-3’-diaminobenzidine (DAB) staining of detached leaves from Ws, mutant 3790, Col-0 and *SKS13* overexpressing transgenic line G101. The arrows indicate the part of each leaf that was injured by forceps to serve as a positive control for the DAB staining.

*SKS13* has a putative function in oxidation/reduction reactions [28,29] and its co-expressed genes function in the generation of reactive oxygen species (ROS) [30,31]. Therefore, we hypothesized that overexpression of *SKS13* may lead to an accumulation of ROS in leaves. To visualize ROS we used 3-3’-diaminobenzidine (DAB) staining on the leaves of Ws, mutant 3790, Col-0 and transgenic line G101 (Figure 4). Each leaf was injured by forceps to serve as a positive control for the DAB staining [32]. In comparison to Ws and Col-0 leaves, darker browning was observed in leaves of mutant 3790 and transgenic line G010, respectively (Figure 4).

### *Brevicoryne brassicae* performance on mutant 3790

It has been suggested that ROS accumulation plays a general role in plant defense against aphids [11,12]. Therefore, we hypothesized that *SKS13* overexpressing plants would not only affect the generalist *M. persicae* but also other aphid species. This hypothesis was tested by infesting mutant 3790 and Ws with the specialist *B. brassicae*. At 14 days after infestation, an average of four *B. brassicae* was found on mutant 3790 and 18 *B. brassicae* on WS plants (Mann–Whitney *U* test *P* < 0.001, n = 15).

### Effect of *SKS13* overexpression on transcription of known JA-, SA- and ET-defense genes

**Figure 5:**
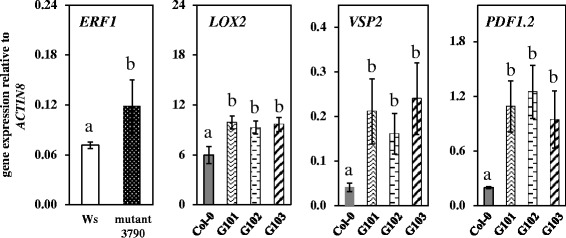
**Expression analysis of ET and JA pathway marker genes in plants without aphid infestation.** Quantitative RT-PCR data are shown for an ET marker gene (*ERF1*) in rosette leaves of Ws and mutant 3790, and for three JA marker genes (*LOX2*, *VSP2* and *PDF1.2*) in rosette leaves of Col-0 and *SKS13* overexpressing transgenic lines G101, G102 and G103. Values are the means ± SD (n = 3). Bars marked with different letters are significantly different from each other within a graph (ANOVA followed by Tukey tests, *P* < 0.05).

To determine whether overexpression of *SKS13* affects the plant hormone pathways known to be involved in plant defense against herbivorous insects, we monitored the expression levels of JA-, SA- and ET-marker genes in mutant 3790, Ws, *SKS13* overexpressing transgenic lines and Col-0 without aphid infestation. In leaves of mutant 3790 the expression levels of the JA-marker genes *LOX2 (Lipoxygenase 2)*, *VSP2* (*Vegetative Storage Protein 2*) and *PDF1.2* (*Putative plant defensin 1.2*) as well as SA-marker genes *PAD4* (*Phytoalexin Deficient4*) and *PR1* (*Pathogenesis-related 1*) were similar as in leaves of Ws (data not shown). However, the expression level of the ET-marker gene *ERF1* (*Ethylene response factor 1*) was significantly higher in mutant 3790 than in WS (Figure 5). Conversely to mutant 3790 (in Ws background), the *SKS13* overexpressing transgenic lines showed significant higher expression levels of the JA-marker genes compared to their corresponding wild type Col-0 (Figure 5). The SA- and ET-marker genes were not affected in these lines (data not shown).

## Discussion

### Overexpression of *SKS13* in leaves enhances resistance to *M. persicae* in *A. thaliana*

Mutant 3790 was previously identified as an *A. thaliana* mutant on which the population development of *M. persicae* was reduced [23] and in the present paper we show that this is, at least partly, due to the constitutive overexpression of *SKS13*. The negative effect of *SKS13* on aphid population development was confirmed in transgenic plants that embraced the *SKS13* under the control of CaMV 35S promoter. An analysis of expression profiles in publicly available microarray data sets revealed that *SKS13* is exclusively expressed in pollen (https://www.genevestigator.com/) [30]. This is in agreement with our observation that *SKS13* was not expressed in leaves of Ws or Col-0. We also demonstrated that the expression of *SKS13* was not induced upon infestation of *M. persicae*. This is consistent with previous microarray studies in which no induction of *SKS13* expression in *A. thaliana* after *M. persicae* infestation was found [33–35].

### Overexpression of *SKS13* affects feeding behavior of *M. persicae* probably due to ROS accumulation

Analysis of *M. persicae* feeding behavior by the EPG technique can provide insight into the plant resistance mechanisms [36]. The EPG results suggest that plant resistance conferred by overexpression of *SKS13* was phloem based. This was supported by the fact that the phloem phase of *M. persicae* on *SKS13* overexpressing plants was delayed in time and reduced in length, while the length of the pathway phase was not significantly different from the control. The phloem based resistance was further indicated by the reduced number of sustained phloem sap ingestions. As sustained phloem sap ingestion is required for the transmission of persistently transmitted viruses [37], the phloem based resistance explains the decreased transmission of such a virus, i.e. *Turnip yellows virus*, as previously observed in mutant 3790 [23].

To uncover the role of *SKS13* in the phloem based plant resistance to *M. persicae*, we explored the possible biological function of this gene*.* As structurally related to multiple-copper oxidases, ascorbate oxidases and laccases, *SKS13* has been suggested to function in oxidation/reduction reactions [28,29]. Furthermore, *SKS13* is co-expressed with genes involved in ROS generation (https://www.genevestigator.com) [30,31]. Therefore we hypothesized that constitutive overexpression of *SKS13* results in an accumulation of ROS in leaves and confirmed this by DAB staining the leaves of *SKS13* overexpressing plants. The effect of ROS accumulation on aphid feeding behavior has also been shown for a triticale cultivar with a high concentration of ROS on which cereal aphids displayed a reduced time in the phloem phase and a prolonged time in the non-probe phase [38]. This is similar to our observations of *M. persicae* feeding behavior on *SKS13* overexpressing plants. The accumulation of ROS was suggested to play a role in plant resistance to several aphid species [11,38]. This is also in line with our results, as aphid resistance on *SKS13* overexpressing plants not only affected *M. persicae* but also *B. brassicae* performance. Besides enhancing aphid resistance, excessive ROS can damage proteins, lipids and nucleic acids and can eventual be harmful to plant growth [39], thereby explaining the reduced size of *SKS13* overexpressing plants.

### Overexpression of *SKS13* affects plant hormone pathways in *A. thaliana*

Several studies suggest that ROS accumulation is linked with the JA, SA and ET plant hormone pathways to play a role in plant defense against aphids [11,12,40,41]. For instance, the *A. thaliana RbohD* mutant, in which JA-induced ROS accumulation does not occur, promotes a four times larger aphid population development than its wild type Col-0 [12,42], suggesting that aphid resistance conferred by activation of the JA pathway is probably mediated by ROS accumulation. In our study, we observed a similar activation of the JA pathway in *SKS13* overexpressing Col-0 plants, as indicated by the significantly higher expression levels of three JA marker genes.

In mutant 3790, *SKS13* is overexpressed in *A. thaliana* accession Ws and the ET pathway is activated instead of the JA pathway, which may be due to the genetic differences between Col-0 and Ws in response to ROS accumulation [43]. Furthermore, ROS may indirectly affect plant growth through altered signaling pathways. Kerchev et al. [44] concluded that a reduced Arabidopsis plant growth results from a low ascorbate, a compound that buffers the production of ROS, that triggers ABA- and JA dependent signaling. As ascorbate buffers the production of ROS, low levels of this compound would result in enhanced ROS accumulation. This is consistent with the observed higher ROS accumulation and reduced plant growth for both mutant 3790 and *SKS13* overexpressing transgenic lines in our study. Our observation that signaling pathways were differently affected suggests that other factors may influence plant growth as well. In addition to *SKS13*, the higher expression of *BRL3* and/or *SKS11* may contribute to this difference. Alternatively the additional differences may be attributed to unknown interactions among BR, ET and ROS. Studying the interaction between *SKS13* and JA-/ET-mediated defense responses may lead to a better understanding of activation of JA and ET responses and their contribution to aphid resistance.

## Conclusions

Overexpression of *SKS13* in *A. thaliana* leads to a reduced phloem feeding of *M. persicae*, which probably is due to accumulation of ROS in leaves. The reduced phloem feeding results in the suppression of the population development of *M. persicae* and also decreases the transmission of persistent viruses. Overexpression of *SKS13* in *A. thaliana* also affects *B. brassicae* and possibly other phloem feeding insects as well. The enhanced resistance towards *M. persicae* and *B. brassicae* in *SKS13* overexpressing *A. thaliana* plants reduces plant development.

## Methods

### Plants

Mutant 3790, was previously identified from an *Arabidopsis thaliana* accession Wassilewskija (Ws) activation tag library as a mutant on which *M. persicae* showed a reduced population development [23]. Seeds of this mutant and its corresponding background accession Ws were obtained from the library present at Wageningen UR Plant Breeding [22]. Seeds of *brl3-2* and *brl3-3* mutants and their corresponding background accession Columbia-0 (Col-0) were kindly provided by Prof. S.C. de Vries, Laboratory of Biochemistry Wageningen University [26]. To induce germination, seeds were placed at 4°C in the dark for 3 days under high humidity. Subsequently, seeds were transferred to potting compost (Lentse Potgrond®) and plants were cultivated in a climate chamber (20 ± 2°C, RH 60-70%, 6 h: 18 h (light: dark). Plants were watered every other day and no pest control was applied. In all experiments we used plants in their vegetative stage, i.e. before they start flowering.

### Insects

*Myzus persicae* (green peach aphid) was reared in cages on Chinese cabbage (*Brassica rapa* L. ssp. *pekinensis* cv. Granaat). *Brevicoryne brassicae* (cabbage aphid) was reared on Brussels sprouts (*Brassica oleracea* L. var. *gemmifera* cv. Cyrus) at the Laboratory of Entomology, Wageningen University. Both rearings were maintained in an acclimatized room at 20 ± 2°C, RH 60-70%, 18 h: 6 h (light: dark). For all experiments, only apterous aphids were used.

### Inverse PCR

Genomic DNA was extracted from leaves of mutant 3790 using the DNeasy Plant Mini kit (Qiagen), digested with restriction enzyme EcoRI (Thermo, product # ER0275) or BamHI (Thermo, product # ER0051) and subsequently ligated with T4 DNA ligase (Fermentas, product # EL0011). Inverse PCR was performed according to the method described previously [21]. PCR products were sequenced and then blasted against the *A. thaliana* genome (http://www.arabidopsis.org/) [45].

### Time course experiment of *M. persicae* infestation

To determine if the *SKS11* and *SKS13* gene are induced upon *M. persicae* infestation, we performed a time course experiment. Four-week-old plants were infested with 15 wingless aphids of assorted life stages. Leaf material was collected after zero, six and 24 hours of aphid infestation. Aphids were gently brushed away from the leaf tissue and uninfested plants were also brushed. Leaf material was immediately flash frozen in liquid nitrogen and stored at −80°C until use.

### Quantitative RT-PCR

Samples were designed in three biological replicates, with 17 individual plants pooled per replicate. Total RNA isolation, cDNA synthesis and quantitative RT-PCR (qPCR) were performed according to the methods described previously [21]. Gene specific primers were designed with Primer-3-Plus software [46] and are listed in Table 2. Threshold cycle (Ct) values obtained with qPCR were normalized for differences in cDNA synthesis by subtracting the Ct value of the constitutively expressed gene *ACTIN8* (At1g49240) [47] from the Ct value of the gene of interest. Normalized gene expression was then calculated as 2^-∆CT^ and Log-transformed prior to analysis. Independent-samples *t*-test or ANOVA followed by Tukey tests were used to determine the significance between genotypes/treatments (*P* < 0.05).

**Table 2:** **Sequences of gene specific primers used for quantitative RT-PCR analyses**

**Gene name**	**Forward primer (5' → 3')**	**Reverse primer (5' → 3')**
*BRL3*	GGACATACCCGGGAGTACCT	CCCGTGTCTCAGATTTTGGT
*SKS11*	CAACTGTGGAATGTGGAACG	GGTGACAAGACACTCGCGTA
*SKS13*	GAGCTACGAAGGAAGCAACG	CACTGGCGGTTAAGTTCCAT
*LOX2*	AGATTCAAAGGCAAGCTCCA	ACAACACCAGCTCCAGCTCT
*VSP2*	TACGAACGAAGCCGAACTCT	GGCACCGTGTCGAAGTCTAT
*PDF1.2*	CACCCTTATCTTCGCTGCTC	GCACAACTTCTGTGCTTCCA
*PAD4*	GTTCTTTTCCCCGGCTTATC	CGGTTATCACCACCAGCTTT
*PR1*	GGCCTTACGGGGAAAACTTA	CTCGCTAACCCACATGTTCA
*ERF1*	CTTCCGACGAAGATCGTAGC	TCTTGACCGGAACAGAATCC
*ACTIN8*	GATGGAGACCTCGAAAACCA	AAAAGGACTTCTGGGCACCT

### Generation of transgenic plants

To generate transgenic lines overexpressing *SKS13*, the coding region fragment of *SKS13* was amplified from Col-0 cDNA using primers AttB1_SKS13_F (GGGGACAAGTTTGTACAAAAAAGCAGGCTCGAGCGAGAGAGATTCAAAAAT) and AttB2_SKS13_R (GGGGACCACTTTGTACAAGAAAGCTGGGTTCCTCTC TGGATTGAACAATGA) in a PCR reaction containing the Phusion™ enzyme (Finnzymes, Product codes: F-530S, 100U). The following PCR program was used: 30 seconds at 98°C followed by 35 cycles of 98°C for 10 sec, 64°C for 10 sec, and 72°C for 3 min with a final extension at 72°C for 10 min. The resulting PCR product was extracted from a 1% agarose gel using the QIAquick Gel Extraction Kit (Qiagen) and sequenced for verification. The verified coding region fragment of *SKS13* was transferred into donor vector pDONR207 using the Gateway® BP Clonase™ II enzyme mix (Invitrogen) to generate entry vector pDONR207::*SKS13*. The entry vector was subsequently cloned into Gateway destination vector pFAST-R02 [48] using the Gateway LR® Clonase™ II enzyme mix (Invitrogen) to generate the expression construct pFAST-R02-*SKS13* in which *SKS13* is under the control of the CaMV 35S promoter. The construct was transformed into *E. coli* and transformants were checked by colony PCR using primers AttB1_F (GGGGACAAGTT TGTACAAAAAAGCAGGCT) and AttB2_R (ACCACTTTGTACAAGAAAGCTG GGT). After verifying the accuracy of the coding region fragment of *SKS13*, the construct was transformed into *Agrobacterium tumefaciens* strain GV3101 [49] by electroporation. *Agrobacterium* mediated transformation [50] was used to introduce the pFAST-R02-*SKS13* plasmid into Col-0 flowers. Seeds containing the construct were selected using fluorescence microscopy (Zeiss, SteREO Discovery.V8) [48].

### No-choice aphid assays

Nymph producing adult aphids (both *M. persicae* and *B. brassicae*) were collected from rearing plants and placed on detached cabbage leaves in a petri dish overnight. New born one-day-old nymphs were placed in the centre of three-week-old *A. thaliana* plants using a fine brush. Each plant received one nymph and the total number of aphids was counted 14 days after infestation. The plants were randomly organized with 15 biological replicates per genotype. Plants were separated by a water barrier to prevent aphids crossing over from one plant to the other. The Mann–Whitney *U* test or Kruskal–Wallis followed by Mann–Whitney *U* test were used to determine if differences between genotypes were significant (*P* < 0.05).

### Electrical penetration graph

The electrical penetration graph (EPG) technique [24] was employed to monitor the feeding behavior of *Myzus persicae*. A gold wire (diameter 20 μm) was attached onto the dorsum of young adult aphids using conductive water-based silver glue. The wired aphid was placed on a mature leaf of a five-week-old plant that was connected to a recording system via a copper electrode in the soil. All tested aphids stayed at the underside of the leaf. The EPGs were recorded at 22°C with constant light for 8 hours. The EPG data were analyzed using the PROBE 3.0 software (Wageningen University, the Netherlands) to distinguish the various waveforms. Waveform C represents the pathway phase, when the aphid stylet is penetrating through the leaf tissue; waveform E2 represents phloem sap ingestion; Waveform F is associated with penetration difficulties; and waveform G indicates active uptake of water from the xylem. Both sequential and non-sequential parameters were analyzed [51] to characterize probing behavior of individual *M. persicae*. At least 15 recordings of individual aphids (one aphid per plant) were obtained for each genotype. The Mann–Whitney *U* and Fisher exact test were used to determine the significance difference between genotypes (*P* < 0.05).

### Determination of reactive oxygen species (ROS) accumulation

To visualize reactive oxygen species (ROS), leaves were cut from four-week-old plants and submerged overnight in an HCl solution containing 1 mg mL^−1^ 3-3’-diaminobenzidine (DAB), pH 3.7 [52]. Chlorophyll was extracted with 96% ethanol overnight at room temperature. Leaves were subsequently photographed in 80% glycerol.
